# Aerobic exercise significantly alters *Notch1* signaling in a neurotoxic hippocampal rat model of Alzheimer's disease

**DOI:** 10.1177/13872877261434058

**Published:** 2026-03-25

**Authors:** Yosra Tavan, Somayyeh Roozegar, Ziya Fallah Mohammadi, Abolfazl Akbari, Khadijeh Nasiri, Mozhgan Memarmoghaddam, Vahid Talebi, Darpan I. Patel

**Affiliations:** 1 Department of Exercise Physiology, Faculty of Physical Education and Sport Science, 48531 University of Mazandaran, Babolsar, Iran; 2 Motor Behavior, 48531 University of Mazandaran, Mazandaran, Iran; 3 Department of Sports Sciences, Faculty of Humanities, 256565 University of Maragheh, Maragheh, Iran; 4 16058 School of Nursing, University of Texas Medical Branch at Galveston, Galveston, TX, USA

**Keywords:** Alzheimer's disease, *HES1* gene, *HEY1* gene, hippocampal, *Notch1* signaling, *RBPJK* gene

## Abstract

**Background:**

Alzheimer's disease (AD) is a progressive neurodegenerative disorder and a major cause of dementia.

**Objective:**

The aim of this study was to investigate the effect of aerobic training on the expression changes of *Notch1*, *Rbpjk*, *Hes1*, and *Hey1*genes in the hippocampus of Alzheimer rats.

**Methods:**

Forty, 8-week-old male Wistar rats were divided into four groups: control (n = 10), exercise (n = 10), AD (n = 10), AD + exercise (n = 10). Endurance training was implemented with increasing intensity starting at 15 m/min in the first week and progressing to speeds of 16–20 m/min over the next five weeks with increased durations each week. After 6 weeks, animals were euthanized and hippocampus was collected, frozen and RNA was isolated to quantify *Notch1*, *Rbpjk*, *Hes1*, and *Hey1* expression. All statistical analyses and graphs were conducted using SPSS and visualized using GraphPad Prism, with significance set at p < 0.05.

**Results:**

One-way analysis of variance with Tukey's post-hoc analysis found that the Alz group had significantly lower expression of *Notch1* with increased expression of *Rbpjk* and *Hes1*. Conversely, the AD + Ex group was observed to have significantly higher *Notch1* and significantly lower *Rbpjk* compared to the AD group.

**Conclusions:**

These findings suggest that exercise may serve as a complementary neuroprotective intervention via manipulation of *Notch1* signaling. Overall, the study highlights the need for further research on the relationship between physical activity and gene expression in the context of AD.

## Introduction

Alzheimer's disease (AD) is the leading cause of dementia and is rapidly emerging as a very costly, deadly, and burdensome neurological disease. Since 2016, significant progress has been made in several areas: our comprehension of the disease's underlying pathology has improved, multiple causative and protective genes have been identified, new blood-based and imaging biomarkers have been discovered, and there are initial promising indications of positive outcomes from disease-modifying treatments and lifestyle changes.^1,2^ Nerve weakness and death of neurons is the main cause of irreversible deficits associated with AD. Since the death of neurons usually occurs in the later stages of this progressive disorder, the cell signaling events that occur before cell death in the early stages of the disease are of special therapeutic importance.^3^

NOTCH is an evolutionarily-conserved single-pass transmembrane receptor that affects numerous cell fate decisions through shortrange cell–cell interactions. NOTCH protein consists of the extracellular domain (NECD) with 29–36 epidermal growth factor (EGF) repeats for ligand binding, the transmembrane domain (TM), and the intracellular domain (NICD) with transcriptional activity.^4^ The *Notch* signaling cascade is critical for development, cell proliferation, differentiation and homeostasis. Aberrant signaling is found in various cancers as well as CNS malignancies.^5,6^ *Notch* signaling is one of the main regulators of the behavior of neural stem cells in specific areas of the brain during development and adulthood.^7^ The *Notch* pathway is a focal cell signaling system that regulates cell fate, differentiation, and tissue homeostasis. *Notch* activity induces hippocampal neuron assembly and regulates synaptic plasticity and memory formation. On the other hand, hyperactivation of *Notch* signaling can cause neuronal death following neuronal injury. In AD brains, *Notch1* signaling is reduced in neurons, suggesting that loss of function in *Notch* signaling may preserve neuronal continuity at the expense of synaptic plasticity, which contributes to the unfolding and progressive memory loss in AD. Dysregulated *Notch* activity has pathological consequences. *Notch* genes encode a group of membrane receptors (*Notch1* to *Notch4* in mammals), with a large extracellular domain and intracellular domain involved in nuclear signaling via membrane-bound ligands (delta-like and serrated/serrate). This process activates *Notch* receptors and leads to nuclear translocation of NCID. In the nucleus, the NCID associates with the effector transcription factor *Rbpjk/Csl.*^8^ Cleavage of this protein associates with the transcriptional repressor *Rbpjk* in the nucleus and leads to the recruitment of activators, turning *Rbpjk* into an activator and creating a complex required for the transcription of downstream targets. So far, only a few related target genes have been found and widely accepted as common targets of *Notch.*^9^ Among others, members of the *Hes* and *Hey* gene families are common targets of the *Rbpjk* community.^10^

Regular exercise has been reported to delay the advancement of cognitive impairment and enhance cognitive function, particularly executive function.^11^ Aerobic activities, specifically, are associated with better performance in daily tasks, improved mental well-being, and a decrease in body-wide inflammation. These exercise programs boost quality of life by preserving physical capabilities, alleviating anxiety and depression, and promoting social engagement; all of which contribute to more effective disease management and a slower worsening of symptoms.^12,13^ Physical inactivity is responsible for 13% of all AD cases and as such, aerobic exercise has been shown to reduce the risk of AD. An increase of only 25% in physical activity can prevent almost one million cases of AD.^14^ Therefore, physical activity has been considered as a supplement to treatment and a possible approach to correcting AD.^2^

Several animal studies have shown the beneficial effects of physical exercise on brain function and health.^2,14^ Regular physical exercise seems to have a protective effect against AD by inhibiting various pathophysiological molecular pathways involved in AD.^15^ Although there are many studies on the positive effect of exercise in different ways of AD,^16,17^ the effect of aerobic exercise on gene expression of *Notch1* and *Rbpjk/Hey, Hes* family, has not been investigated. Therefore, this research aims to answer the following questions: Does aerobic training influence the changes in *Notch1, Rbpjk/Hey, and Hes* gene expression in the hippocampus of AD mice?

## Methods

**Figure 1.**
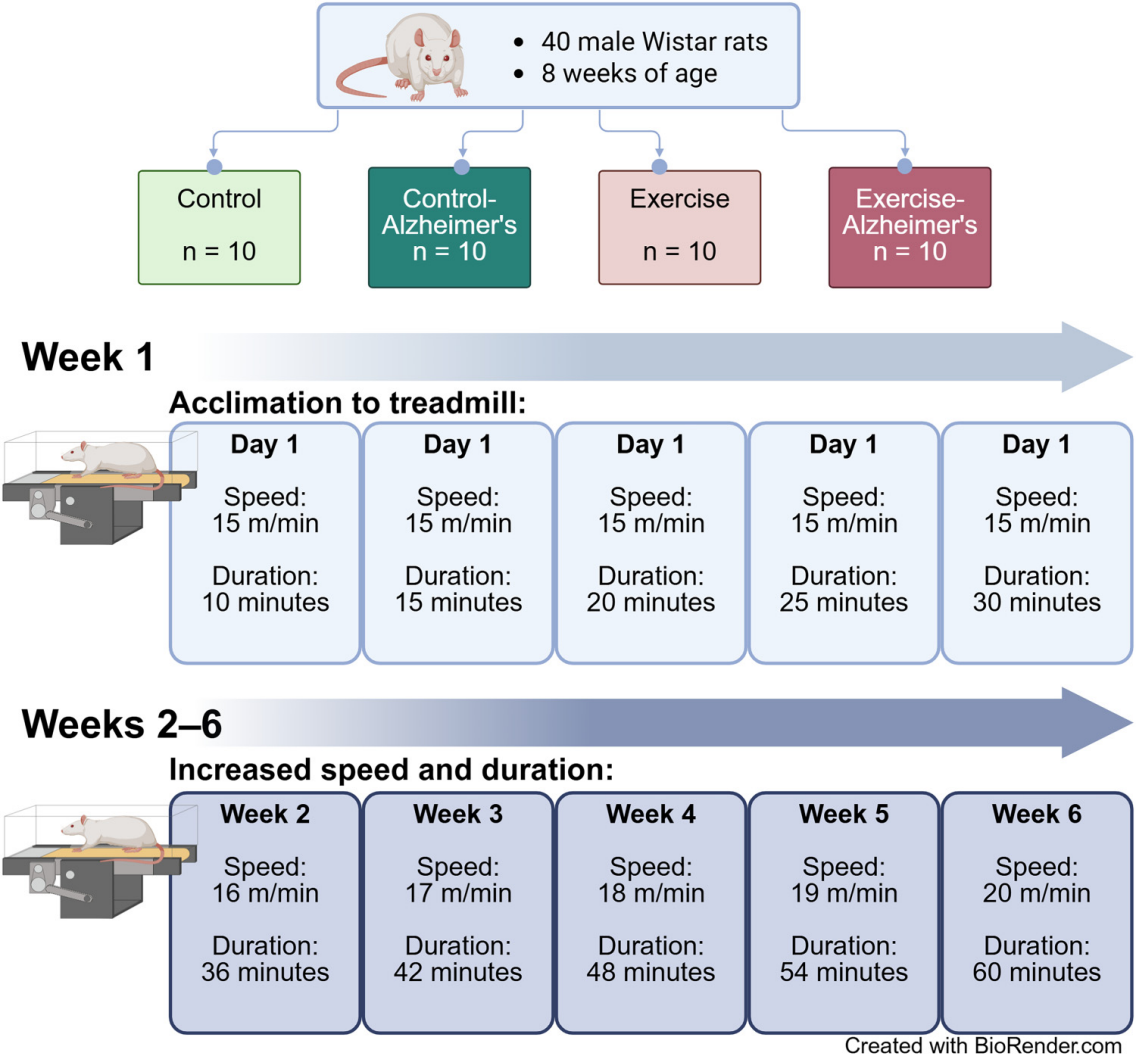
Study design. Forty male rats were randomized to one of four treatment groups. Rats in one of the two exercise groups completed an acclimation protocol in week 1 followed by 5 weeks of progressive treadmill exercise. Animals in the control groups maintained normal activity with standard enrichment.

Forty, 8-week-old male *Wistar* rats were utilized in this 4-group, randomized, post-test only study design (Figure 1). Only male *Wistar* rats were included to ensure experimental stability and minimize confounding variables associated with sex-specific hormonal fluctuations. After a 1-week acclimatization period, animals were randomly divided into one of 4 groups: Control (Con: n = 10), AD (Alz: n = 10), Exercise (Ex: n = 10), AD + Exercise (Alz-Ex: n = 10). The study has 80% power to detect a between-group difference with an effect size of d = 0.80 at a two-sided alpha of 0.05, using a one-way ANOVA framework with post hoc comparisons as needed. All experiments were performed in accordance with the guidelines provided by the animal laboratory and according to the approval received from the Institutional Animal Care and Use Procedures at the University of Mazandaran (Approval : IR.UMZ.REC.1401.076).

### Alzheimer's disease induction

Trimethyltin (TMT; 0.8 mg/kg) was injected intraperitoneally to the two AD groups (Alz and Alz-Ex).^18^ Animals were monitored for 17 days after. On day 17, TMT treated animals were evaluated for AD conditions using the shuttle box test and the Morris water maze (MWM) test. Equivalent volumes of 0.9% saline were injected into the non-AD groups as a sham. We employed the TMT-induced neurotoxicity model because it is widely recognized for producing selective hippocampal neuronal loss, oxidative stress, and cognitive deficits. Although the TMT model does not fully replicate the amyloid-β or tau pathology characteristic of AD, it effectively mimics hippocampal degeneration and memory dysfunction that occurs during the early neurotoxic phase of AD.^19–21^ Therefore, this model provides a reliable and reproducible experimental platform for investigating neuroprotective interventions such as exercise, which target hippocampal vulnerability and neuronal plasticity in AD-like conditions.

### Behavioral assessments of Alzheimer's disease

#### Passive avoidance learning test (shuttle box)

The shuttle box test was carried out using a 20 × 80 × 20 cm box, which has a light chamber part (safe area) and a dark chamber part (unsafe area). separated by a sliding door. The bottom of the dark chamber is equipped with rods that transmit an electric current with an intensity of one milliamp and a frequency of 50 Hz for 3 s. Inhibitory avoidance method was performed to check memory in laboratory mice in 2 consecutive days.

#### Memory retrieval test

Twenty-four hours after the training, the retrieval test was performed to check the long-term memory of the animal. At this stage, each animal was placed in the bright side of the chamber and after 20 s the sliding door was opened and the time it took for the animal to enter the dark side was recorded. The experiment was terminated when the animal entered the dark chamber. The experiment was terminated if animals remained in the light chamber for 5 min. The delay time for the animal to step into the dark compartment during the recovery phase was recorded as a measure of memory.

#### Morris water maze (MWM) test

The MWM test is a behavioral tests used to assess spatial learning and memory.^22^ For this test, rodents were placed in a circular metal tank filled with water, made to be opaque by the black walls of the tank, with a dark metal platform submerged 1–5 cm. The walls were lined with geometric shapes to assist the rodents to navigate the location of the platform. The movement and behavior of the animal was tracked by means of an infrared camera placed two meters above the central part of the tank. This method collects the time it took for the animal to find the Plexiglas platform, the length of the swimming path, the percentage of time the animal spends in each quarter of the tank, and movement speed. Room lighting was adjusted to avoid reflections interfering with video recording or automated data acquisition.

### Exercise protocol

The exercise intervention consisted of 6-week treadmill exercise performed 5 times per week. The treadmill apparatus was a 10-chamber system with a 90 cm long belt. Rats were familiarized to the treadmill in week 1 of training beginning with 10 min of activity in day 1 to 30 min of activity in day 5. Speed was maintained at 15 m/min during this period. Speed in weeks 2–6 ranged from 16–20 m/min for a duration of 36–60 min, progressively increasing speed and duration each week. This protocol was modified only in duration from a previously published design.^23^ See Figure 1 for details.

### Euthanasia and tissue processing

Seventy-two hours after the final training session, or the final day of the intervention period for control animals, rats were anesthetized by intraperitoneal injection of a combination of Ketamine (50 mg per kg) and Xylazine (4 mg per kg) as previously described.^24^ Euthanasia was performed by decapitation. To collect the hippocampus samples, the skull was split using a surgical blade and the brain was carefully removed. The brain was divided into two halves exactly in the middle and the hippocampal sinus was separated from the limbic system with the help of a clean atlas. The hippocampus samples were collected and immediately frozen with liquid nitrogen and stored in a freezer at −80°C until analysis.

### RNA isolation and sequencing

RNA was extracted from hippocampus tissue using a commercially available RNA extraction kit (Denazist Zist Asia). Then, cDNA was synthesized with oligo dT primer and reverse transcription enzyme. Specific primers for the amplification of *Notch1, Rbpjk, Hes1, Hey1,* and *β-Actin* reference gene were designed using Primer Premier 5 software and then the sequence of primers were synthesized by Bioneer (South Korea). Relative quantity of each gene was done using real time PCR. During this step, polymerase chain reaction was performed for cDNA samples for genes and the reference gene Beta-actin using the SYBR Gren kit from Ampliqon (Denmark) in the Rotor gene Corbett 6000 machine. After checking the values related to the threshold cycle (Ct) obtained from the biological and technical repetitions of each treatment, the average CT was calculated for the technical repetitions of *Notch1, Rbpjk, Hes1, Hey1,* and *β-Actin* genes, and then the data were expressed by the expression ratio of each of the genes. The above specificity was calculated for *β-Actin* (reference gene). The expression level of the desired genes was calculated by the Livak method (2-ΔΔCT). Primers are presented in Table 1.

**Table 1.** Genes and primer sequence.

Genes	Primer sequences	Sizes (bp)
Hey1	F-5′-CTGGCTGAAGTTGCCCGTTA-3′	103
	R-5′- GCTGGGATGCGTAGTTGTTG-3′	
Rbpj	F-5′-GTTTTTTTGCCCACCTCCTT-3′	123
	R-5′-CCTATCCCAATAAATGCACACG-3′	
Hes1	F-5′-CACAGAAAGTCATCAAAGCCTATC-3′	165
	R-5′-GAGGTGCTTCACTGTCATTTCC-3′	
Notch1	F-5′-TGTCATCTCCGACTTCATCTATC-3′	112
	R-5′- CTTGGCAGCATCTGAACGA-3′	
β-Actin	F-5′-GTGTGACGTTGACATCCGTAAAGAC-3′	119
	R-5′-TGCTAGGAGCCAGGGCAGTAAT-3′	

### Statistical analysis

The Shapiro-Wilk test was used to check the normality of the data, and the Lune test was used to check the assumption of equality of variances. To statistically analyze the data and to confirm the AD of mice, t-test was used, and one-way variance (ANOVA) test and Tukey's post hoc test were used to compare differences between the research groups. Data are presented as mean ​​±standard deviations.

## Results

### Behavioral assessment outcomes confirm Alzheimer's disease induction

Table 2 presents the outcomes of the shuttle box test and Morris water maze tests that confirm the onset of AD symptoms in treated rodents.

**Table 2.** Body mass and behavioral assessment outcomes.

Groups	Shuttle box test (seconds)	MWM completion time (seconds)	MWM distance traveled (meters)	MWM velocity (meters/seconds)
Control (n = 20)	152.5 ± 29.79	92.25 ± 19.5	16.7 ± 5.0	20.9 ± 5.6
Alzheimer's disease (n = 20)	36.9 ± 11.7***	112.4 ± 20.5***	21.2 ± 7.4*	17.9 ± 4.6*

*p < 0.05; ***p < 0.001.

### Notch1 expression is preserved with exercise

**Figure 2.**
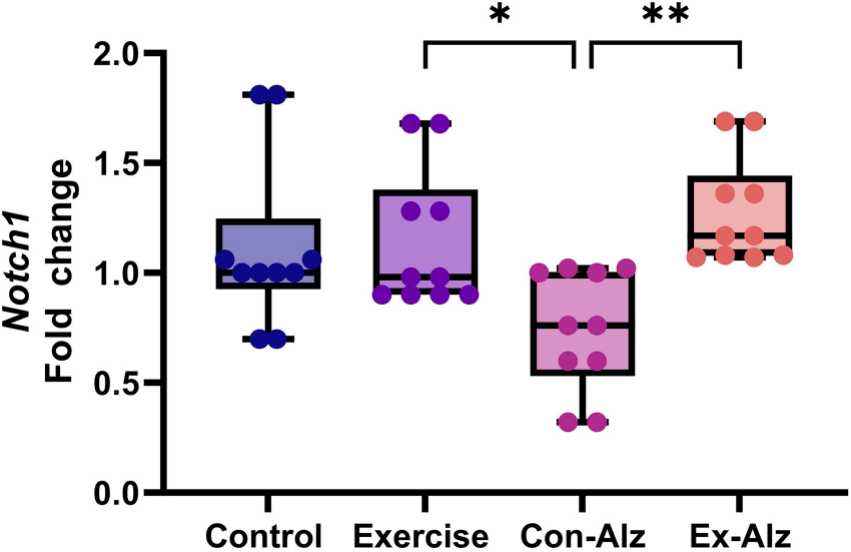
Comparison of hippocampal *Notch1* gene expression of research groups after six weeks of progressive training. Significant difference between the Exercise group (1.15 ± 0.3) and the Con-Alz group (0.74 ± 0.3; p = 0.05). **Significant difference was observed between exercise-Alzheimer's group (1.27 ± 0.2) and Con-Alz (0.74 ± 0.3; p = 0.03).

The results of the one-way analysis of variance showed that there is a significant difference between the groups regarding *Notch1* gene expression (F(3, 36) = 5.441, p = 0.003). Marked reduction on *Notch1* expression was observed between the Con-Alz group and the other three treatment groups. Exercise was shown to attenuate this loss of *Notch1* expression on the Alz-Ex group, showing similar expression to the Exercise group (Figure 2).

### Hes1 expression is high in Alzheimer's disease

**Figure 3.**
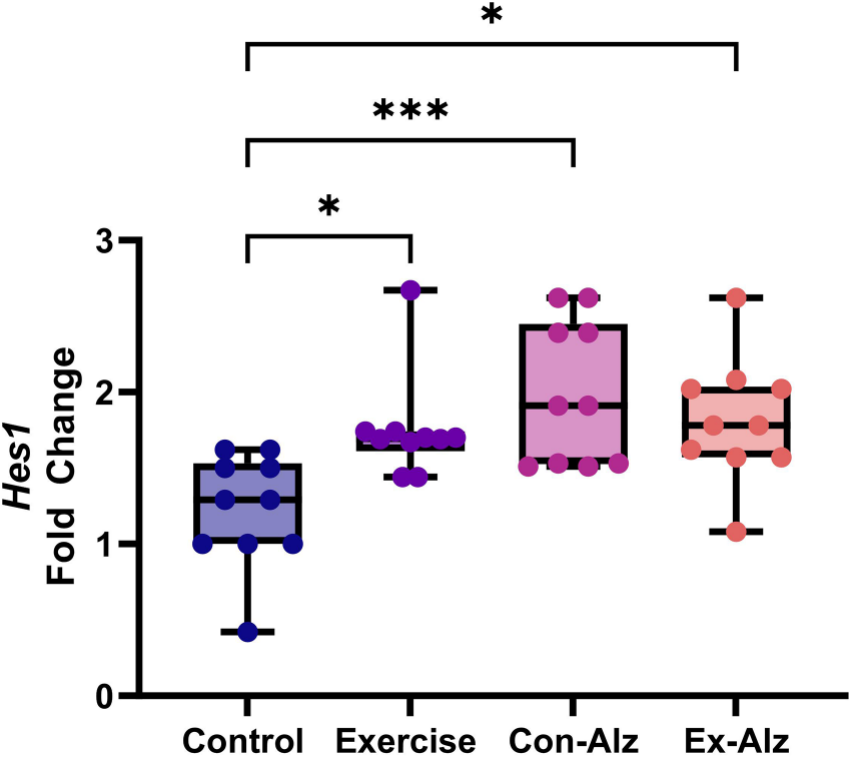
Comparison of hippocampal *Hes1* level of research groups after six weeks of progressive training. Control (1.22 ± 0.4) animals had significantly lower concentration compared to exercise (1.75 ± 0.3; p = 0.03)*, Con-Alz (1.99 ± 0.5; p < 0.001)*** and Ex-Alz (1.8 ± 0.4; p = 0.01)*, respectively.

There was a significant difference between the groups regarding *Hes1* gene expression (F(3, 36) = 6.691, p = 0.001). The results of Tukey's post hoc test showed that there is a significant difference between the control group and other groups (p < 0.05). No difference was observed between the exercise and AD groups, respectively (Figure 3).

### Rbpjk expression is reduced with exercise

**Figure 4.**
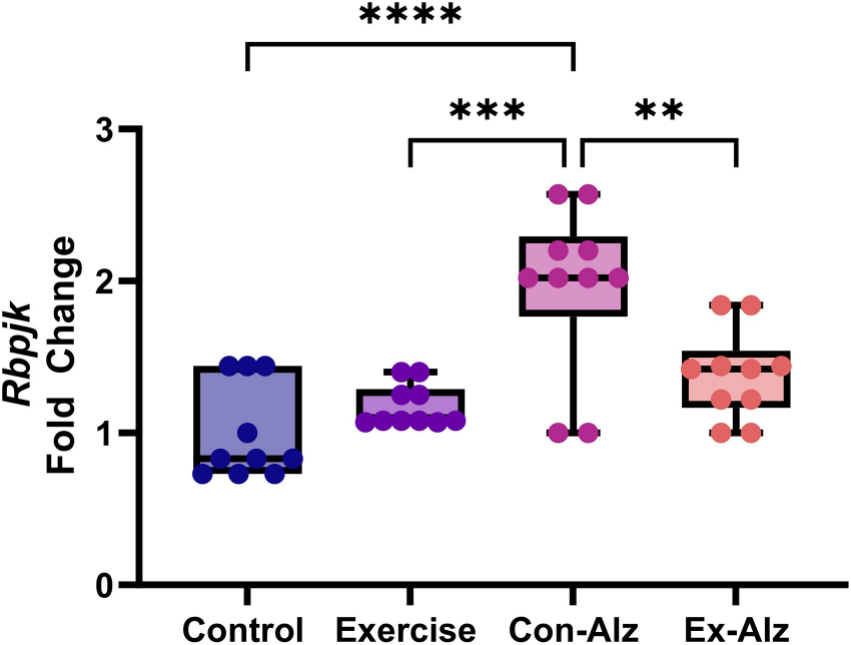
Comparison of *Rbpjk* hippocampal level of research groups after six weeks of progressive training. The Con-Alz (1.9 ± 0.5) group had significantly higher expression compared to controls (1.0 ± 0.3; p < 0.001), exercise (1.2 ± 0.1; p < 0.001) and Ex-Alz (1.34 ± 0.5; p = 0.004), respectively.

The one-way ANOVA found significant differences between the groups regarding *Rbpjk* gene expression (F(3, 36) = 13.886, p < 0.001). Tukey's post hoc test results showed that *Rbpjk* expression was significantly higher in the Con-Alz group compared to all other groups (p < 0.01). Exercise in the AD group led to a maintenance of *Rbpjk* expression similar to non-AD groups, respectively (Figure 4).

### Hey1 expression remains unchanged with exercise

**Figure 5.**
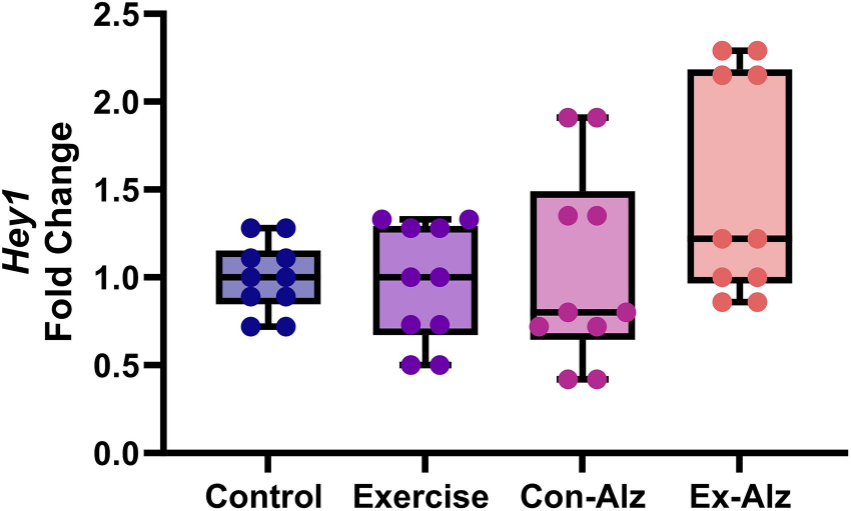
Comparison of hippocampal *Hey1* level of the research groups after six weeks of progressive training. No significant difference was observed between Control (1.0 ± 0.2), Exercise (0.97 ± 0.3), Con-Alz (1.04 ± 0.6) and Ex-Alz (1.5 ± 0.5) group, respectively.

Significant difference between the groups regarding *Hey1* gene expression (F(3, 36) = 2.954, p = 0.045) was observed (Figure 5). However, in the post-hoc analysis, no significant differences between groups were found.

## Discussion

The purpose of this study was to examine the effects of aerobic exercise on *Notch1* signaling in AD rats. In summary, despite a non-significant change in *Notch1* expression, *Rbpjk* and *Hes1* were significantly increased in the AD model. Exercise was able to stabilize *Notch1* signaling while also maintaining *Rbpjk* expression, though *Hes1* expression remained to be significantly increased. In conventional canonical *Notch1* signaling, any changes in *Notch1* expression should be met with similar changes in *Rbpjk* and *Hes1*. In our study, we found an inverse effect in AD animals, such that a decrease in *Notch1* was met with an increase in *Rbpjk* and *Hes1* gene expression. This raises the possibility of alternative explanations associated with noncanonical signaling due to oxidative stress or neuroinflammatory damage. Exercise was able to stabilize *Notch1* and *Rbpjk* to levels similar to controls providing course correction. *Hes1* remained elevated, suggesting a potential acute stimulus effect of exercise. While evidence suggests that exercise can positively influence AD-related gene expression, further studies are needed to elucidate the specific mechanisms by which exercise impacts *Notch1* and its downstream effects on cognitive function.

*Notch1* signaling pathway plays a significant role in various brain functions, including synaptic plasticity and neurogenesis. In the context of AD, studies have shown that *Notch1* expression is significantly altered. Specifically, *Notch1* accumulates in fibrillary plaques and neurofibrillary tangles, which are hallmarks of AD. This accumulation is consistent with decreased *Notch1* expression and activation in neurons, suggesting a dysfunctional signaling pathway that may contribute to disease-related cognitive decline.^25,26^ As stated earlier, canonical signaling would suggest similar changes in gene expression in both *Rbpjk* and *Hes1* to that of *Notch1*. The results of this study suggest noncanonical influence that may be driving the observed levels of gene expression. Potential explanations center around neuroinflammatory injury and AD severity. In neural cells, *Hes1* can be activated independent of the *Notch/Rbpjk* complex through other pathways like JNK, Hedgehog and Wnt.^27–29^ Activation of these alternative pathways can directly upregulate *Hes1* transcription even when levels of *Notch1* are low.^30^ AD severity is also associated with changes in *Notch* targets, particularly during the intermediate stages of AD. This may likely be a neuroprotective response exhibited in the hippocampus of the AD rats prior to the decline typically seen in the advanced stages of AD.^30^

Research highlights exercise as a powerful intervention to improve cognitive function and potentially alter gene expression related to AD. Exercise has been identified as a favorable intervention for reversing gene expression patterns associated with AD, including those associated with *Notch1.*^31–33^ Our results suggest that exercise in our TMT-induced neurotoxicity model was effective in rescuing *Notch1* expression and *Notch1*'s signaling cascade as evident by the normalization of *Rbpjk* expression. *Hes1* overexpression in the exercise models may be associated with growth factor crosstalk due to increased expression of brain-derived neurotrophic factor (BDNF) and TrkB.^34–37^ Methi et al. (2024) showed that exercise training leads to increased *Hes1* levels.^38^ *Hes1* is known to have protective effects against neurotoxic agents such as Aβ, which are implicated in AD. *Hes1* overexpression can counteract the deleterious effects of Aβ on neuronal survival and morphology, suggesting its potential as a therapeutic target.^39^ Modulation of *Hes1* expression by exercise offers several implications for AD patients. Increased *Hes1* expression through regular physical activity may support cognitive health by promoting neurogenesis and protecting against Aβ toxicity.^39^ Continued research into the molecular mechanisms underlying these effects is necessary to better explain the noncanonical response observed in this study.

The results of the study regarding *Hey1* expression showed that there is no significant difference between all groups. Moderate-intensity exercise has been shown to improve neural function, particularly in the hippocampus, contradicting the results of this study. Previous work has found that increased *Hey1* is associated with increased levels of BDNF and it signaling pathways, which may affect the expression of various genes.^37^ Modulation of these processes may involve changes in gene expression profiles, including those related to synaptic plasticity and neurogenesis.^37,40^ While direct evidence linking *Hey1* expression specifically to exercise in AD is still emerging, the broader implications of physical activity on neuroprotective gene regulation are well supported by other research.

### Limitations and strengths

The results of this study should be interpreted in the context of the limitations to this study design. It is important to note that the TMT-induced neurotoxicity model primarily reflects hippocampal neuronal damage and does not encompass the full spectrum of amyloid-β or tau-related pathology observed in clinical AD. Thus, the current findings should be interpreted within the framework of hippocampal neurodegeneration rather than a complete AD phenotype. Nevertheless, this model remains a valuable tool for exploring mechanisms of neuronal injury and protection, particularly in relation to exercise-mediated modulation of oxidative stress, inflammation, and neuroplasticity in the hippocampus. Future studies incorporating transgenic or amyloid/tau-based models could further validate and extend our findings. The rationale for selecting the TMT model is its reproducible induction of selective hippocampal damage, oxidative stress, and memory impairment, which make it a suitable and widely accepted tool for investigating neuroprotective and exercise-mediated interventions targeting hippocampal vulnerability in AD-like conditions.^19–21^ Another limitation to this study is the lack of protein level data. Ideally, the inclusion of western blot or immunodetection of full-length NOTCH1, Hes1, and RBP-Jκ would help better explain if changes in gene expression are translated to changes in protein expression.

### Conclusion

The results of our study found that while *Notch1* expression was downregulated following TMT exposure, *Hes1* levels were elevated, suggesting the presence of feedback or non-canonical mechanisms within the *Notch* signaling network. These results corroborate previous studies that have reported that *Hes1* can be upregulated through *Notch*-independent pathways or via autoregulatory loops that respond to cellular stress and inflammatory cues. Therefore, the observed *Hes1* increase in our study may reflect an adaptive mechanism aimed at maintaining neuronal homeostasis under conditions of neurotoxic stress and *Notch1* suppression. These findings highlight the complexity of the *Notch1–Rbpjk–Hes1* regulatory axis and suggest that exercise may modulate this pathway indirectly by influencing broader molecular networks involved in neuronal resilience.

## Supplemental Material

sj-xlsx-1-alz-10.1177_13872877261434058 - Supplemental material for Aerobic exercise significantly alters *Notch1* signaling in a neurotoxic hippocampal rat model of Alzheimer's disease Supplemental material, sj-xlsx-1-alz-10.1177_13872877261434058 for Aerobic exercise significantly alters *Notch1* signaling in a neurotoxic hippocampal rat model of Alzheimer's disease by Yosra Tavan, Somayyeh Roozegar, Ziya Fallah Mohammadi, Abolfazl Akbari, Khadijeh Nasiri, Mozhgan Memarmoghaddam, Vahid Talebi and Darpan I. Patel in Journal of Alzheimer's Disease
